# Supplementary material for: Structural Basis for Childhood Antibody Recognition of The Human Metapneumovirus Fusion Protein
Source: bioRxiv. 2025 Sep 3:2025.08.29.673069. Preprint. [Version 1] doi: 10.1101/2025.08.29.673069 (PMC12424945; doi:10.1101/2025.08.29.673069)
Supplement: Supplement 1 [file media-1.pdf]

Table S1. Metadata for the monoclonal antibodies isolated from hMPV-infected children.				
mAb	Participant ID	Participant Sex	Participant age at infection (year)	Repeated infection (Yes/No) *
MPV498	9131	Male	1	No
MPV499	8749	Male	1	No
MPV510, MPV511 and MPV513	8631	Female	1	No

*This refers to whether the infection is repeated during the cohort (2011 to 2016).

Table S2. Monoclonal antibody sequences.

mAb	HC amino acids	HC Junction	LC amino acids	LC Junction
MPV498	QVQLQESGPGLVKPSETLSLTCTVSGGSISSYYWSWIRQPAGKGLEWIGRIYTSGNTNYNPSLKSRVTMSLDTSKNQVSLKLSSVTAADTAVYYCARSRVATTPVGLRDWLDPWGQGTLVTVSS	CARSRVATTPGLRDWLDPW	TPSLSASVGDRVTITCRASQSISSYLNWYQQKPGKAPKLLIYAASSLQSGVPSRFSGSGSGTDFTLTISSLQPEDFATYYCQQSYSTPLFFGQGTKLEIK	CQQSYSTPLFF
MPV499	QVQLQESGPGLVKPSQTLSLTCTVSGGSISSGDYNWNWIRQHAGKGLEWIGYINYSGSTDYNPSLKSRVTISVDTPKNQFSLKLTSVTAADTAVYYCARGVDFWSGYCDYWGQGSLVTVSS	CARGVDFWSGYCDYW	PVSLSASVGDRVTITCRASQSISTYLNWYQQKPGKAPKLLIYAASSLQSGVPSRFSGSVSGTDFTLTISSLQPEDFATYYCQQTYTTPLTFGGGTKVEIK	CQQTYTTPLTF
MPV510	QVQLQESGSGLVKPSQTLSLTCAVSGGSISSGDSSWSWIRQPPGKGLEWIGHVYESGNTYYDPSLQSRVTISVDRSRNQFSLKLTSVTVADTAVYYCAREGNYGWDYFDYWGQGTLVTVSS	CAREGNYGWDYFDYW	DIQMTXSPFSLSASVGDRVTITCRASQSINSYLNWYQQKPGKAPRLLIYAASSLQSGVPSRFRGSGSGTDFALTISSLQPEDFATYYCQQSYRPPSRTFGQGTKVEMK	CQQSYRPPSRTF
MPV511	QVQLQQWGAGLLKPSETLSLTCGVDGGSFNGYYWNWVRQLPGKGLEWIGEVSYAGSDNYNPSLKSRATISGDRSRKQFSLRLDSVTVAGTGVYYCARDKGVLDYSFGLDVWGQGTTVTVSS	CARGKGVLDYSFGLDVW	DIQMTQSPSTLSASVGDRVTITCRASQSVSNWLAWYQQKSGKAPKLLIYKASSLESGVPSRFSGSGSGTEFTLTISGLQPDDFATYYCQEHNSDSRAFGQGTKVEIK	CQEHNSDSRAF
MPV513	EVQLVQSGAEVKKPGESLRISCKGSGYDFPSYWISWVRQMPGKGLEWMGRIDPTDSNTNYSPSFQGHVTLSADKSISTAYLQWSSLKASDTAIYYCARHSDSWSYYEDSWGQGTLVTVSS	CARHSDFWSYYEDSW	DIQMTQSPSSLSASVGDRVTITCRASQSISTYLNWYQQKPGKAPKLLIYAASSLQSGVPSRFSGRLSGTDFTLTISSLQPEDFATYYCQQTYSSPRTFGQGTKVEIK	CQQTYSSPRTF

**Table S3. Cryo-EM data collection, refinement, validation, and model-building statistics.**				
	MPV498/hMPV B2-post fusion F	MPV499/hMPV DsCav-ES2 pre-fusion F	MPV510/hMPV DsCav-ES2-IPDS pre-fusion F	MPV513/hMPV DsCav-ES2 pre-fusion F
**Data collection and processing**				
Scope	Titan Krios	Titan Krios	Titan Krios	Titan Krios
Magnification	59,000	59,000	59,000	105,000
Voltage (kV)	300	300	300	300
Electron exposure (e–/Å^2)	60	60	60	60
Defocus range (μm)	0.8-2.8	0.8-2.8	0.8-2.8	0.8-2.8
Camera	DE-Apollo	DE-Apollo	DE-Apollo	Gatan K3
Pixel size (Å)	0.79	0.79	0.79	0.88
Symmetry imposed	C3	C1	C3	C1
Initial particle images (no.)	2,500,000	150,000	1,200,000	1,800,000
Final particle images (no.)	111,000	22,000	287,000	208,000
Map resolution (Å)<br>FSC threshold	2.61<br>0.143	6.57<br>0.143	2.88<br>0.143	3.94<br>0.143
Map resolution range (Å)	2-6	5.5-10	2.3-7	3.5-6
**Refinement**				
Initial model used (PDB code)	7M0I/Alphafold	N/A	7UR4/Alphafold	6W16/Alphafold
Model resolution (Å)<br>FSC threshold	2.61<br>0.143	N/A	2.88<br>0.143	3.94<br>0.143
Model resolution range (Å)	2-6	N/A	2.3-7	3.5-6
Map sharpening *B* factor (Å^2)	87.5	452	105.2	234.9
R.m.s. deviations<br>Bond lengths (Å)<br>Bond angles (°)	 0.002<br>0.521	N/A	 0.004<br>0.680	 0.003<br>0.602
Validation<br>Clash-score<br>Poor rotamers (%)	 6.12<br>2.83	N/A	 24.21<br>3.37	 6.49<br>3.29
Ramachandran plot<br>Favored (%)<br>Allowed (%)<br>Disallowed (%)	 96.55<br>3.45<br>0.00	N/A	 92.78<br>6.14<br>1.08	 91.53<br>7.97<br>0.50
PDB accession code	9OS5	N/A	9PDY	9PDX
EMDB accession code	EMD-70793	EMD-72382	EMD-71548	EMD-71547
EMPIAR accession code	TBD	TBD	TBD	TBD

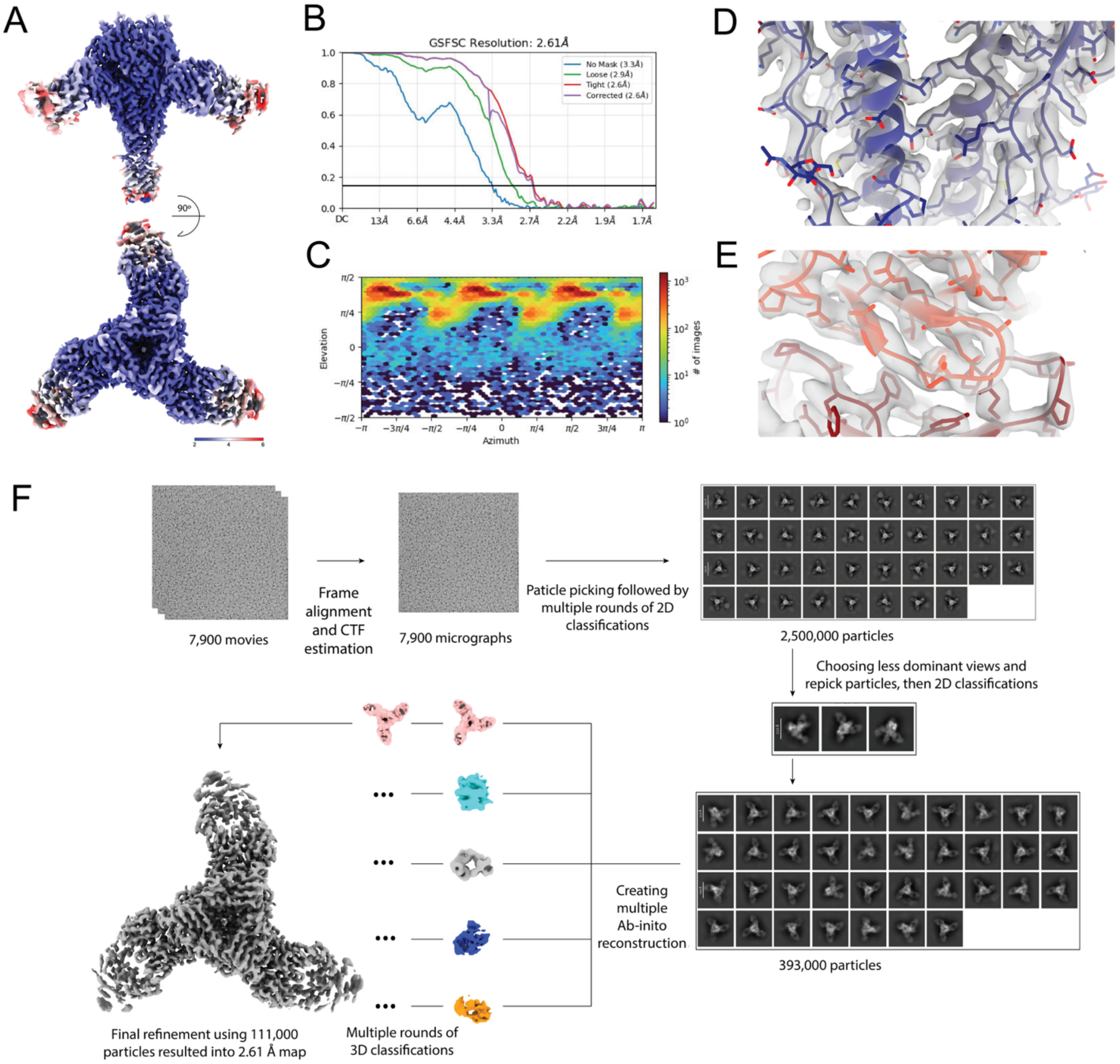

Figure S1. MPV498 cryo-EM processing workflow. (A) Local resolution map obtained for hMPV B2 post-fusion F protein bound to the MPV498 Fab. (B) GSFSC curve of the refined Cryo-EM map. (C) Particle distribution map for the final refinement. (D) Model to map fit example of the hMPV B2 post-fusion F protein. (E) Model to map fit example of the MPV498 Fab near the binding site. (F) Overall Cryo-EM data processing workflow.

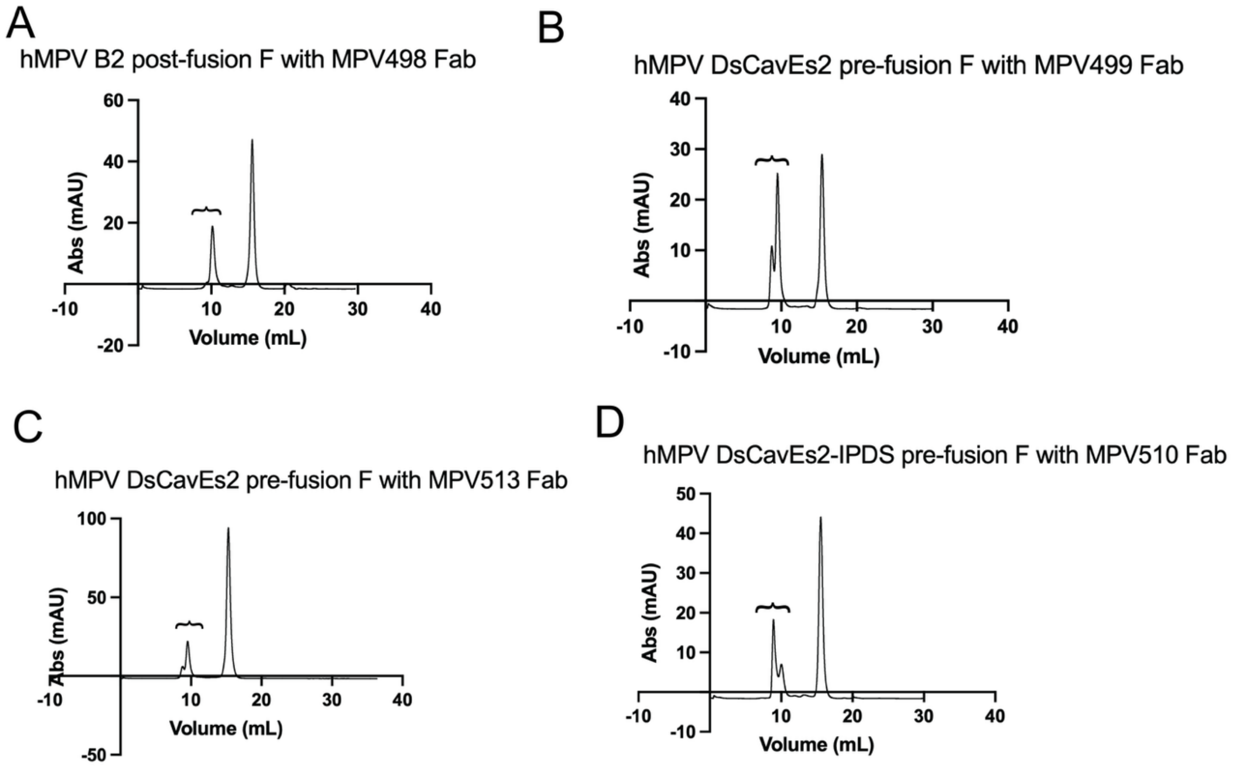

Figure S2. Size-exclusion curves obtained from hMPV F-Fab complexes. (A) hMPV B2 post-fusion F with MPV498 Fab. (B) hMPV DsCavEs2 pre-fusion F with MPV499 Fab. (C) hMPV DsCavEs2 pre-fusion F with MPV513 Fab. (D) hMPV DsCavEs2-IPDS pre-fusion F with MPV510 Fab. Selected complex peaks used for grid preparation are shown with accolades.

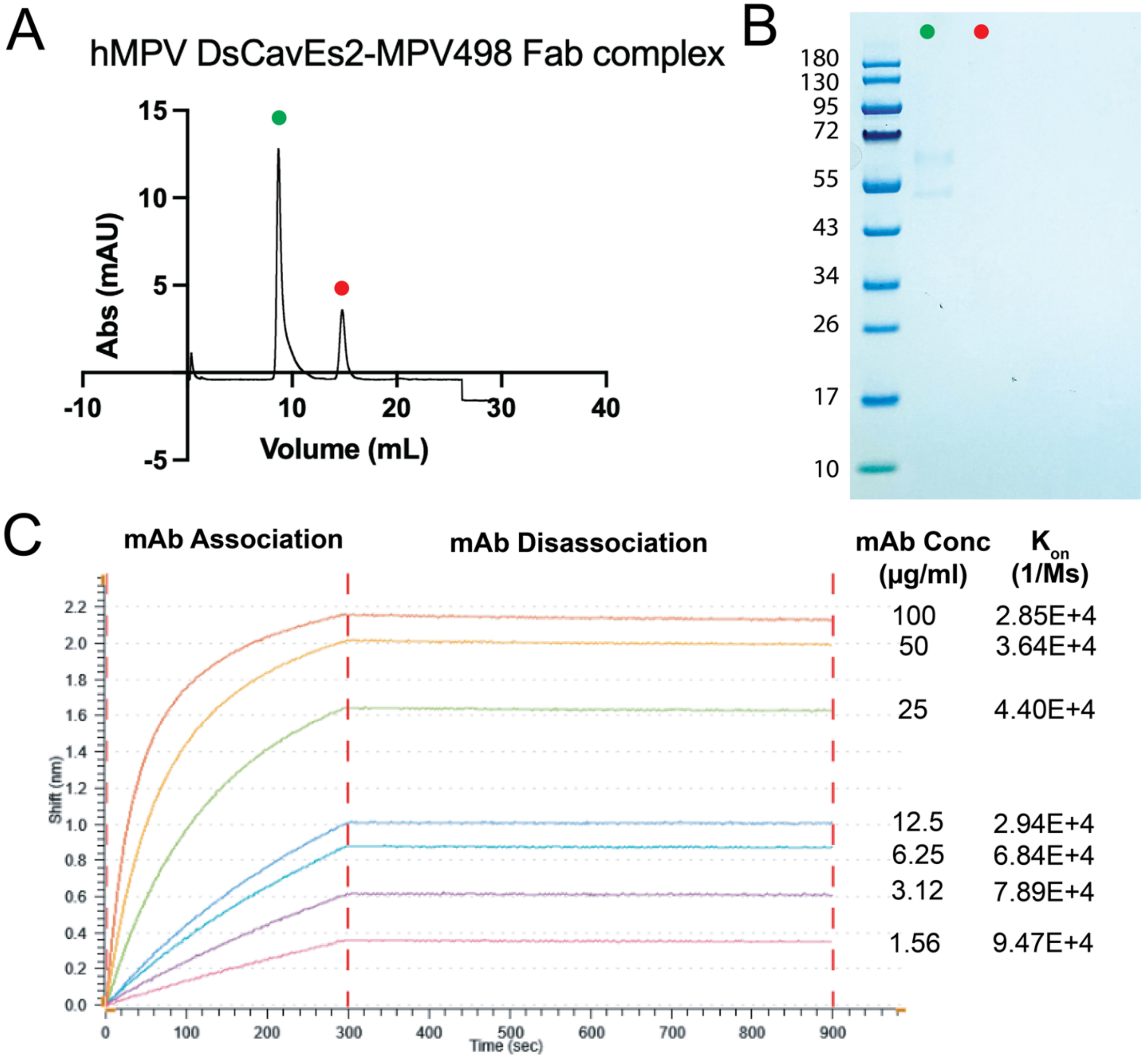

Figure S3. Binding of MPV498 to hMPV DsCavEs2 F. (A) Size exclusion curves obtained from AKTA-pure for hMPV DsCavEs2 F-MPV498 Fab complex (green) and extra Fab (red). (B) SDS-PAGE for the hMPV DsCavEs2 F-MPV498 Fab complex (green) and extra Fab (red). (C) Binding affinity of mAb MPV498 to DsCavEs2 F using BLI. hMPV DsCavEs2 protein (100 μg/ml) was loaded onto anti-His biosensors. Processed curves for the associations (300 s) and disassociations (600 s) of 2-fold serially diluted mAb MPV498 (starting with 100 μg/ml) are shown. K_{on} values are listed for each mAb MPV498 dilution. A reference curve containing buffer only was subtracted from each value.

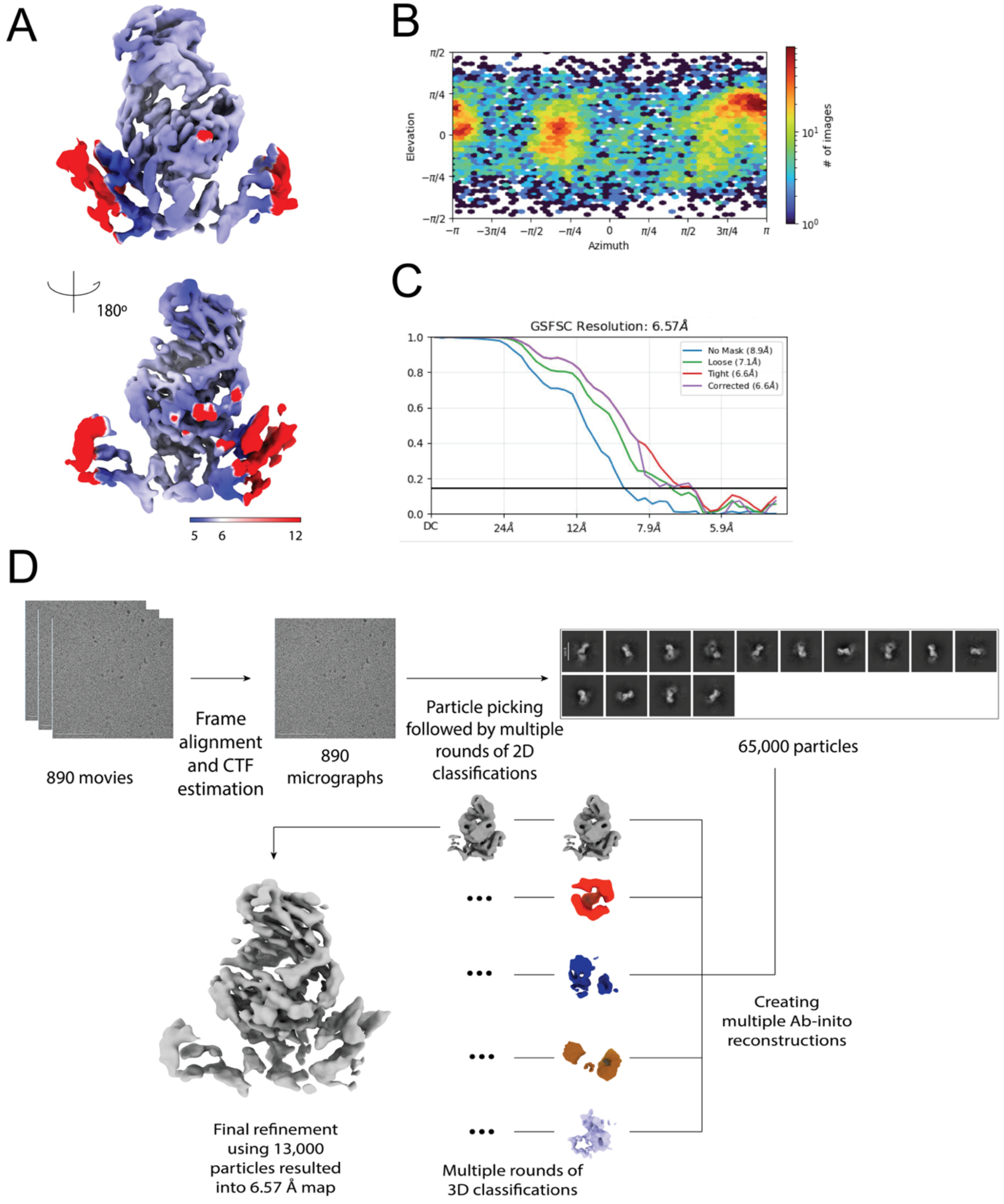

Figure S4. MPV499 cryo-EM workflow. (A) Local resolution map obtained for hMPV DsCav-ES2 pre-fusion F protein bound to the MPV499 Fab. (B) GSFSC curve of the refined Cryo-EM map. (C) Particle distribution map for the final refinement. (D) Overall Cryo-EM data processing workflow.

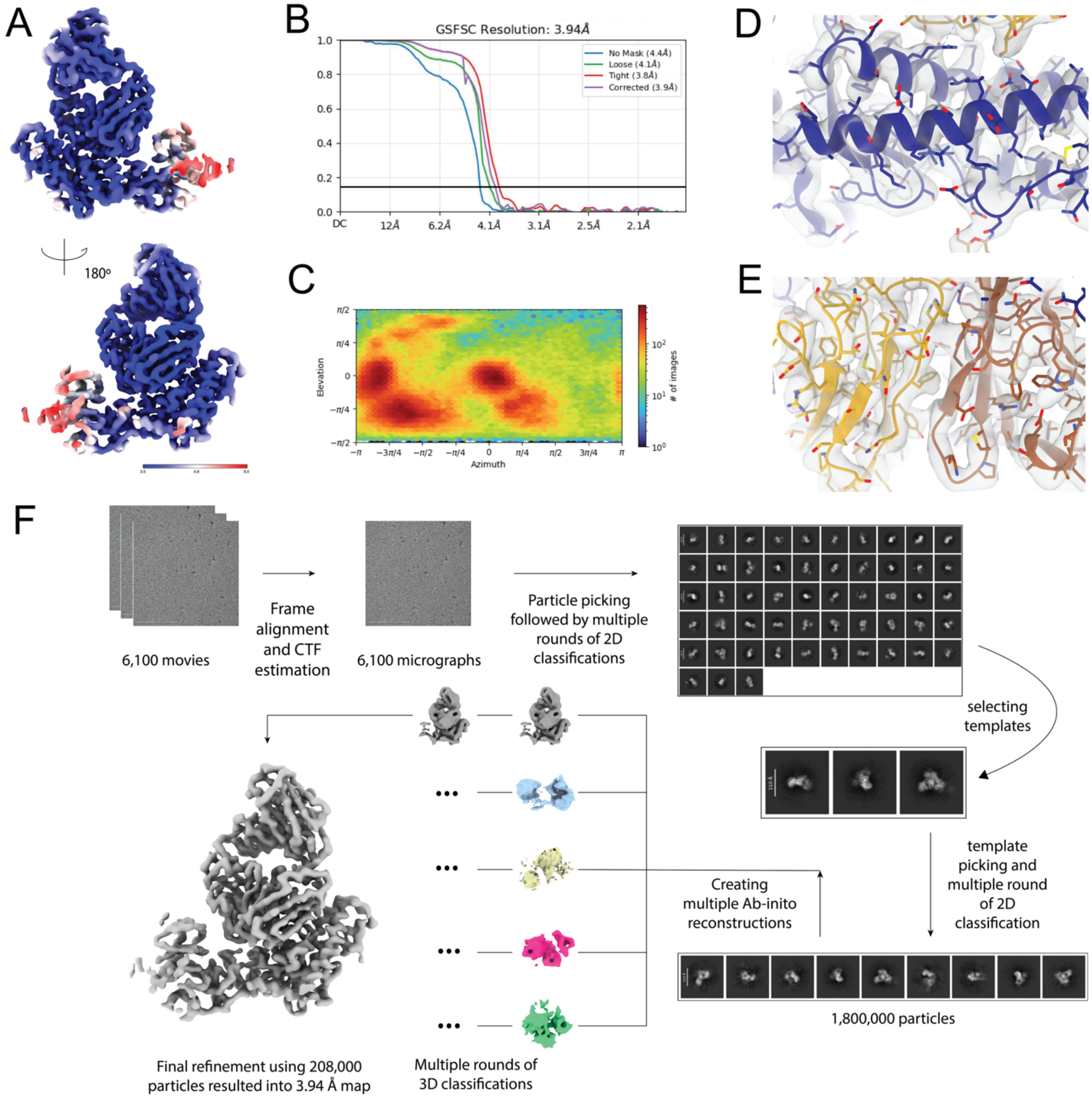

Figure S5. MPV513 cryo-EM workflow. (A) Local resolution map obtained for hMPV DsCav-ES2 pre-fusion F protein bound to the MPV513 Fab. (B) GSFSC curve of the refined Cryo-EM map. (C) Particle distribution map for the final refinement. D) Model to map fit example of the hMPV DsCav-ES2 pre-fusion F protein. (E) Model to map fit example of the MPV513 Fab. (F) Overall Cryo-EM data processing workflow.

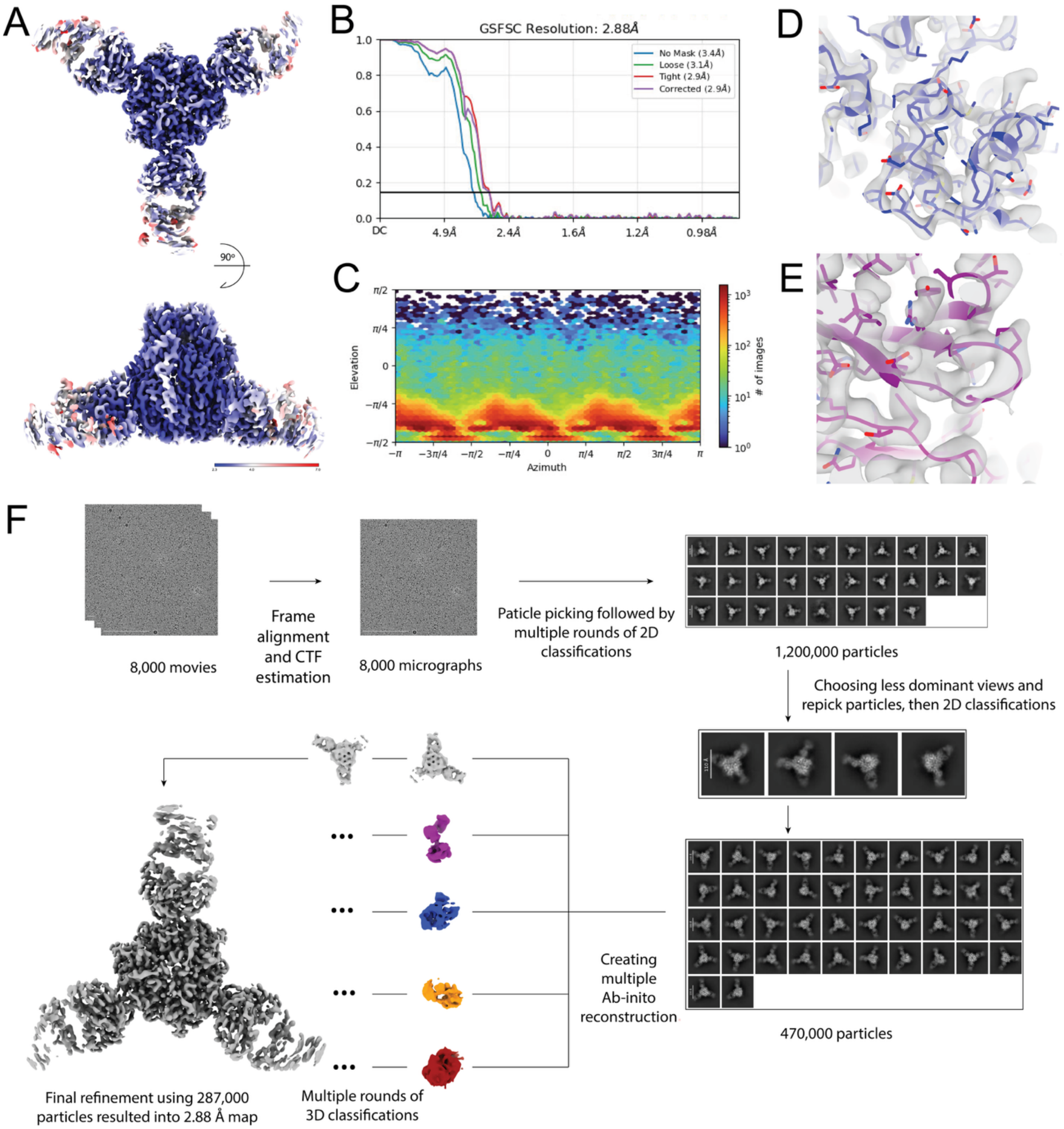

Figure S6. MPV510 cryo-EM workflow. (A) Local resolution map obtained for hMPV DsCav-ES2-IPDS pre-fusion F protein bound to the MPV510 Fab. (B) GSFSC curve of the refined Cryo-EM map. (C) Particle distribution map for the final refinement. (D) Model to map fit example of the hMPV DsCav-ES2-IPDS pre-fusion F protein. (E) Model to map fit example of the MPV510 Fab. (F) Overall Cryo-EM data processing workflow.
